# The rare and the common: scale and the genetic imaginary in Alzheimer's disease drug development

**DOI:** 10.1080/14636778.2019.1637718

**Published:** 2019-07-16

**Authors:** Richard Milne

**Affiliations:** 1 Wellcome Genome Campus – Society and Ethics Research Group, Cambridge, UK; 2 Institute of Public Health, University of Cambridge, Cambridge, UK

**Keywords:** Alzheimer’s disease, genetics, drugs, scale, models

## Abstract

In this paper I examine how the promissory value of genetics is constituted through processes of scale and scaling, focussing on the relationship between “rare” and “common” forms of disease. I highlight the bodies and spaces involved in the production of post-genomic knowledge and technologies of Alzheimer's disease and the development of new disease-modifying drugs. I focus on the example of the development of a monoclonal antibody therapy for Alzheimer's disease. I argue that the process of therapeutic innovation, from genetic studies and animal models to phase III clinical trials, reflects the persistent importance of a genetic imaginary and a mutually constitutive relationship between the rare and the common in in shaping visions of Alzheimer's disease medicine. Approaching this relationship as a question of scale, I suggest the importance of attending to how and where genomic knowledge is “scaled” or proves resistant to scaling.

## Scale and the promise of genomic medicine

In Alzheimer's disease as elsewhere, realizing the therapeutic contribution of post-genomic knowledge has been conceptualized as a problem of “translation.” This involves a bumpy and oft-thwarted movement along a fundamentally linear trajectory between “bench and bedside” (Harrington and Hauskeller 2014; Latimer 2013). However, translation is also fundamentally a question of the ability to scale. Take, for example, Bill Clinton's famous comments at the completion of the Human Genome Project, which emphasized how “In coming years, doctors increasingly will be able to cure diseases like Alzheimer's, Parkinson's, diabetes and cancer by attacking their genetic roots” (Clinton 2000). Such interventions establish hierarchies which link societal health challenges to molecular “roots,” reflecting and reinforce material and functional connections between genes and populations. The politics of scale is thus central to “big biology” (Davies, Frow, and Leonelli 2013), and the process of switching scales, and the forms of knowledge, power and value associated with this a key site of empirical analysis.

In this paper I examine the role of scale and the challenges of scaling in establishing the promissory value of genetics. I focus on how the relationship between the bodies and spaces associated with “rare” and “common” forms of disease is established and configured through the development of new drugs. Focussing on the case of solanezumab, a candidate monoclonal antibody therapy for Alzheimer's disease, I highlight how the interaction and calibration of the rare and the common supports the continuing importance of a genetic imaginary in drug development in AD.

## Background

Alzheimer's disease has, throughout its history, been an unstable category, and the relationship between dementia and biological change continually contested (Ballenger 2006; Lock 2013). At the heart of these debates has been the question of whether “diseases of old age” can be distinguished and treated separately, or whether they represent, and should be managed as, “normal ageing” (Moreira 2017). However, less considered is the relationship between early-onset forms of Alzheimer's disease, which are often familial and genetically inherited and the sporadic, late-onset forms. Focussing on this relationship provides a means of outlining important dynamics in the pursuit of novel treatments for Alzheimer's disease, and contributing to wider discussions about the role of genetics in post-genomic biomedicine.

In the early 1990s, Lippman proposed what has become the classic version of the geneticization thesis – that genetic explanations would come to dominate how societies understand health, illness and social problems (Lippman 1992). Drawing on earlier critiques of medicalization, she critiqued the role of molecular genetics and its institutional and commercial forms in fostering reductionist approaches which obscured complex social and biological processes. Geneticization in this form was, Lippman argued, a process of “colonization” (1992, 1474) explicitly linked to corporate interests.

Geneticization has been the focus of much research interest and debate (Hall 2003; Hedgecoe 2001; Kerr 2000; Shostak, Conrad, and Horwitz 2008; Weiner and Martin 2008; Will, Armstrong, and Marteau 2010). Reflecting on this work, Weiner *et al.* (2017) argue that few initial concerns about geneticization have been realized. They suggest that even for those conditions understood as genetic, genetic knowledge is not privileged over other clinical information. Lock uses Alzheimer's disease to demonstrate this point, arguing that even when a gene is incontrovertibly associated with susceptibility to a disease – in this case the ApoE E4 allele – it does not follow that a genetic mode of interpretation will become dominant across the sites where the condition is investigated, treated, or managed (Lock 2013; Lock, Lloyd, and Prest 2006).

Lock's analysis of Alzheimer's disease is indicative of an emerging “postgenomic consensus” (Navon and Eyal 2016, 1417) which has in turn challenged sociologists to innovate in their understanding of the social consequences of genetic science. Increasing complexity and dynamism in scientific accounts of disease have been accompanied by more nuanced social scientific work which captures relationships between bioscientific, clinical and lay understandings of health, illness and heredity (Arribas-Ayllon, Bartlett, and Featherstone 2010; Weiner and Martin 2008; Will, Armstrong, and Marteau 2010).

Three themes in this recent work have importance for the current discussion. First, while early critical work on genetics presupposed a linear trajectory from science to clinic and society, there is increased recognition of complexity and the non-linearity of socio-technical change. Shostak, Conrad, and Horwitz (2008) argue that relations between genetics research, geneticization and medicalization are “path-dependent.” Drawing on the examples of depression, homosexuality and chemical exposure, they argue that longer-term socio-technical histories and decisions made at critical junctures within these establish enduring institutional and structural patterns of classification and categorization. For example, they suggest that the attribution of genetic causes to depression is the result of “cultural definitions, institutional forces, and political and economic interests that arose decades ago” (2008, S304). In contrast to homosexuality then, the geneticization *and* medicalization of depression are rendered possible by the prior location of the phenotype within the “jurisdiction” (2008, S304) of medicine.

These socio-technical legacies and paths of classification are important in considering what Weiner *et al.* describe as the persistence of a “genetic imaginary,” rooted in a fundamentally biological understanding of disease and the promise that “[b]y understanding the molecular basis of disease we can create a new kind of medicine.” (2017, 999). While this imaginary, they suggest, is continually remade and rearticulated, it retains a power and attraction aligned with a contemporary focus on pharmaceutical solutions to illness, and, we might expand, the “biomarkerization” (Metzler 2010) of disease. Weiner *et al.* suggest that this genetic imaginary is expressed in a new wave of novel biotechnologies, including gene editing, whole genome sequencing and biological drugs.

Recent work has explored the relationship between the “rare” and the “common” proposed within imaginaries of genomic promise. This work shows how genetic explanations fail to gain traction (Will, Armstrong, and Marteau 2010) or, following Mol (2003), how they contribute to elaborating the ontological multiplicity of conditions such as coronary heart disease (Weiner and Martin 2008). Work by Navon and Eyal (2016) on autism, however, suggests an unfolding historical relationship through which categories such as “rare” and “common” conditions emerge. Drawing on Hacking’s (2007) description of the “looping effects” of social categories, Navon and Eyal describe how genetic evidence helped bring together the diagnostic scope of autism with rare forms of developmental disorder not previously understood as associated with the condition. Subsequently, this extension to new domains of application spurred further refinement and elaboration of diagnostic criteria themselves.

In sum, recent work on geneticization suggests complex relations between genetic, social and clinical contexts across which hopes and fears about genetic knowledge and value are realized. Assemblages (Arribas-Ayllon 2016) of genomic promise emphasize complexity and contingency. They also draw attention to the interaction between both the sites of genomic knowledge, such as the lab and the clinic, but also its scales.

Scale is a long-standing concern of work in the “sociology of translation,” notably the relations between the “big” and “small” worlds (Callon, Lascoumes, and Barthe 2009) and the processes through which size and scale are produced (Callon and Latour 1981). This work emphasizes that scales at which and about which knowledge is produced do not pre-exist translation, but rather are precarious achievements brought into being through processes and practices of “scaling” (Davies, Frow, and Leonelli 2013; Latour 2013). These processes involve “negotiations, intrigues, calculations. acts of persuasion and violence” which enable some actors to speak or act in the place of others (Callon and Latour 1981, 279) and which form the “glue” in chains of translation (Williams-Jones and Graham 2003, 275).

Understood in these terms, the relationship between the rare and the common is not simply a relationship between existing categories, but one between two scales which are emergent within a specific moment in the history of Alzheimer's disease. They are stabilized and brought into relation by processes of translation. Further, the capacity to relate scales or move between them is the key operation in the assembly of actor-networks (Latour 2013). Extending the point in her analysis of contemporary capitalism, Tsing suggests that the ability to change scales is the hallmark of modern knowledge and of understandings of progress (Tsing 2012).

Work on the role of animal models in biological research provides an illustration of how scales are produced and brought into relation in the production of post-genomic knowledge. Animal models enable complex phenomena to become tractable scientific objects, simplifying and standardizing the phenomenon under study (Lewis *et al.* 2013). In doing so they transform the topology of disease, providing simplified and standardized biological forms for the production of knowledge which can be transferred between species or scientific fields (cf Ankeny and Leonelli 2011), and its topography, as standardized “bio-objects” which are themselves circulated within scientific networks of exchange (Davies and Rosengarten 2012; Eriksson 2012; Milne 2016). In the process of reducing biological scale, they are thus able to expand the scope of scientific investigation. Thus Davies, in a discussion of the commodified mouse model describes how animals are brought into both “intimate geographies of corporeal equivalence with humans, and expansive geographies of global translational research” (2012, 126).

The ability of topologies of equivalence to support a global system of research makes them an obligatory passage point in the realization of the “genetic imaginary.” They allow the transfer of knowledge generated in one context or organism to another. Models support the ability of research questions to shift between scales, from the molecular to the population, and facilitate the relations of equivalence and exchange integral to the production of genomic value.

However, as Latour and Tsing point out, scaling requires effort, not least when impediments are encountered. Such impediments are familiar features of discussions of the translational role of animal models and the scales between which they mediate. Models’ ability to work across scales requires significant and ongoing work. This is characterized by “constant iteration and transformation” (Davies and Rosengarten 2012, 132) between model organisms and the phenomena they purport to model (Huber and Keuck 2013; Lewis *et al.* 2013; Nelson 2018). Lewis and colleagues (2013) elaborate on this in discussing the use of animal models in practice in the neuroscience laboratory. The inaccessibility of the workings of the brain poses a challenge for neurological modeling, forming an “epistemic void” (Nelson 2018, 12) around which knowledge is constructed. Drawing on ethnographic work across laboratory sites, Lewis and colleagues examine how models come to be considered as “good enough” to speak for the phenomenon they represent. They show how models operate in two directions, such that:

[S]cientists calibrate animals against the medical phenomena which they are intended to represent. In turn, human medical conditions and the patients who manifest them have to be calibrated against the rodent models. (Lewis *et al.* 2013, 776)

Their notion of “calibration” emphasizes the reciprocal material transformations that occur in the process of establishing relations of similarity or equivalence across scales, and provides a productive resource for thinking about the interactions between the rare and the common in the pursuit of therapies for Alzheimer's disease.

In the remainder of this paper, I develop on this work to consider the iterative relationship between the rare and the common in Alzheimer's disease. I first set out the historical relationship between familial and sporadic forms of Alzheimer's disease, and the role of changing diagnostic criteria in establishing these as distinct but related entities. I then examine the role of genetic forms of disease in shaping understandings of Alzheimer's disease in the 1980s, and the translation of human genetic information into mouse models. I address the work involved in scaling Alzheimer's disease, and highlight the impediments which emerge and which prompt the recalibration of the rare and the common.

## Methods

The paper draws on two sources. The first is the genealogical tracing of the materials of Alzheimer's drug development – specifically solanezumab, and the models, bodies and knowledges with which it is entangled – through the research and gray literature, conference proceedings and transcripts, patents and popular publications. This starts from the unsuccessful EXPEDITION3 trial described above, and works back to the early 1980s, through the first publications of preclinical studies, the development of the models in which these were conducted and foundational work in the molecular biology of Alzheimer's disease. I then retraced the story of the molecule that became solanezumab to the present to examine where, how and by whom connections are made between models, materials and populations. Papers were identified through co-authorship and citation patterns, but primarily by following the materials and models involved in the drug development process. In this approach, the scientific literature and associated commentary are treated as “informants,” in which the nature of life is depicted, emplotted and problematized (Kelty and Landecker 2009). In this sense, scientific papers, and their discussions of materials and methods serve to describe experiments, to make an argument for the basis for claims and orient the field around particular concerns or approaches to their resolution.

The second empirical source is ethnographic fieldnotes from observation at Alzheimer's disease research conferences. These include the Clinical Trials in Alzheimer's Disease conference (CTAD), in San Diego in December 2016, and the Alzheimer's Association International Conference (AAIC) in London in July 2017. These are two of the major annual events in Alzheimer's disease research and drug development. Conferences are important sites for the study of science. They represent the history and future of disciplines are enacted, remembered and planned (González-Santos and Dimond 2015). However, conferences serve different purposes for different audiences. The two conferences focussed on here are oriented towards audiences which are complementary but not entirely overlapping. CTAD is a US-European initiative established in 2009. The conference has around 1000 attendees, and sits at the intersection between academic research and industrial drug development, with a focus on new clinical trials and trial methods. Plenary and parallel sessions in the conference halls are complemented by a panoply of small closed meetings, at which large and small companies interact with academic scientific and medical advisors and those who run their trials at public and private sector clinics. AAIC is the largest conference in Alzheimer's disease research, with around 6000 attendees. Organized and managed by the US-based Alzheimer's Association since 2000, the conference is also primarily focussed on biomedical science and clinical applications, but with a wider scope. Again, there is a large biopharmaceutical industry presence, with smaller meetings supplemented by an exhibition hall of company booths and corporate-sponsored satellite events.

There is no standardized approach to the study of conferences (González-Santos and Dimond 2015). The evidence presented in the current paper takes the form of statements made in highly public forums – and, in the case of CTAD, also webcast on the conference website. Analysis of these public statements was informed by informal conference conversations with researchers from private and public sector institutions. These are not presented verbatim, but provided important context for understanding the development of Alzheimer's research. Formal interviews were also conducted with academic and industry representatives, for which informed consent was sought. This work was reviewed by the University of Cambridge Humanities and Social Sciences ethics committee.

## Scaling Alzheimer's disease

The following sections describe the evolving relationship between the rare and the common in the quest for new drugs for Alzheimer's disease. I start with a discussion of the unsuccessful Phase III clinical trial of solanezumab in 2016, and its role in consolidating the molecular redefinition of Alzheimer's disease. I position this in the longer-term evolution of the Alzheimer's diagnosis and the growing emphasis on biomarkers and complexity in Alzheimer's disease research. I then consider the role of molecular genetic research and animal models in supporting the promise of biomarkers, and in “recalibrating” the relationship between animal models, rare diseases and common, late-onset forms of Alzheimer's disease.

## Doing “real” clinical trials

At the *Clinical Trials in Alzheimer's Disease* conference in San Diego in December 2016, a panel of experts from academia, industry and patients’ organizations convened to discuss the meaning and implications of Eli Lilly's EXPEDITION3 trial of the monoclonal antibody drug solanezumab. The summary results of the study had been released two weeks earlier, revealing that the drug did not meet its primary endpoint – a statistically significant change in cognitive decline in people with mild dementia. The CTAD panel, however, provided the forum for the full release of the results by lead investigator Lawrence Honig. It drew a capacity audience to the 2000-seat hotel ballroom venue, a level of interest reflecting the animating absence of the “cure” in Alzheimer's disease research.

Solanezumab is a potentially “disease-modifying” drug for Alzheimer's disease, one that affects the progression of disease to slow or prevent further cognitive decline. Like most drugs under development, solanezumab focuses on one proposed mechanism of disease aetiology, known as the amyloid cascade hypothesis (Cummings *et al.* 2016). The focus of research since the 1990s, such drugs differ from available pharmacological treatments which alleviate symptoms by boosting levels of neurotransmitters. Modifying disease course has become the central quest of biomedical research into Alzheimer's disease. It has, to date, been quixotic. As a commentary in the *BMJ* concluded, solanezumab's lack of success was not a surprise, as “Alzheimer's disease is the graveyard of drug discovery” (Hawkes 2016, i6389).

However, EXPEDITION3 was novel not only in terms of the drug, but the population upon whom it was tested. It was the first major trial to shift the ground for inclusion from the cognitive to the biological. In addition to meeting clinical diagnostic criteria, all those who took part in the trial had to be “positive” for beta- amyloid protein, a biomarker assessed through imaging with the radiotracer florbetapir (known as Amyvid, and also owned by Eli Lilly), or by levels of beta-amyloid in cerebrospinal fluid taken by lumbar puncture. Neurologist Bruno Vellas, co-chair of the CTAD conference, suggested that this fact made the EXPEDITION3 trial qualitatively different from all the previous efforts of the field, as the first “real trial with Alzheimer's disease patients” (author notes). Maria Carrillo, the chief scientific officer of the Alzheimer Association, described the trial “as [the] first of the next generation of smart Alzheimer trials,” the “first one in which everyone is positive for and screened for the protein that you’re trying to treat” (author notes).

Vellas and Carrillo's commentaries reflect an importance shift in biomedical approaches to Alzheimer's disease, and the place of solanezumab within this. As Greene describes, “pharmaceuticals have become central agents in the definition of disease categories” (2007, 225). The presentation of EXPEDITION3 and the elaboration of “real trials” in Alzheimer's disease draws attention to the central role of biomarkers in contemporary Alzheimer's disease research and in the emerging “proto-platform” (Gardner 2017) of practices and technologies associated with the definition, diagnosis and treatment of the condition. Carrillo's reference to the “protein that you’re trying to treat” highlights the importance of beta-amyloid as more than a biomarker. As I explore in subsequent sections, since the 1980s beta-amyloid has been considered by many as a key component of Alzheimer's disease pathogenesis, but remains a site of considerable contestation.

## Scaling Alzheimer's disease

The disease described by Alois Alzheimer in 1907, as a pre-senile form of dementia characterized by neuropathological evidence of “plaques,” “tangles” and the loss of neurons, was formally held separate from common “senility” until the 1950s. Historian Jesse Ballenger suggests that this distinction reflected the general lack of interest and low stakes in an underdeveloped clinical field, and that, while Alzheimer's disease was “officially” a rare form of pre-senile dementia, “every researcher of this period seriously engaged in the study of dementia was aware that Alzheimer's disease and senile dementia were for all practical purposes the same entity.” (Ballenger 2006, 46).

From the 1970s, this practical classification was formalized as Alzheimer's came to be understood as a common and leading cause of illness and death. This was facilitated by an assemblage of techniques of electron microscopy, clinical reclassification and an emergent social movement which engaged political interest in diseases of ageing, drawing together familial and late-onset disease (Ballenger 2006; Fox 1989). One of the architects of this shift, National Institute of Aging director Robert Katzman, reflected later that the goals of a pivotal 1977 research meeting had been to change the scale of both the research effort and of the disease itself:

[t]o reach consensus that Alzheimer disease (AD) was not just a relatively rare neurodegenerative disorder of the presenium, but was the major cause of dementia in the elderly in developed countries. (Katzman and Bick 2000, xi)

The scaling of disease spurred the elaboration of the first diagnostic criteria to define its bounds. The 1984 NINCDS-ADRDA or McKhann criteria (McKhann *et al.* 1984) represented a provisional stabilization of knowledge and practice whose value was predicated on its ability to support and incorporate novel clinical and research insights (Moreira 2017). The criteria set out definitions for both probable and possible Alzheimer's disease based on the presence of dementia. However, they failed to address a central problem of clinical research in Alzheimer's disease, the heterogeneity of the patient population. As the US Office of Technology Assessment described in 1987:

Diagnostic uncertainty complicates clinical research by mixing patients with different diagnoses. A drug or diagnostic procedure maybe tested on patients with disparate diseases. Those with different illnesses or in different stages may respond but be undetected because they are lost among a large group of patients who show no effect. … Patient heterogeneity is thus the bane of efficient clinical testing … There is no clear way around this problem in clinical research on dementia. (OTA 1987, 89)

In the 1990s, criteria for “mild cognitive impairment” (MCI) attempted to provide a remedy by capturing “subtle” and “early” (Petersen 2004, 183) aspects of a cognitive transition between normal ageing and dementia, and providing a new “conventional standard” for the articulation of clinical and research practice (Moreira, May, and Bond 2009). More recently, a comprehensive revision of diagnostic guidelines was proposed by the National Institute of Ageing and the Alzheimer Association (NIA-AA) in 2011 (Lock 2013). These covered clinical applications in dementia (McKhann *et al*. 2011) and MCI (Albert *et al.* 2011), and moved “towards defining the preclinical stages of Alzheimer's disease” (Sperling *et al.* 2011). The NIA-AA criteria suggest a trajectory of cognitive but also biological change, and means of tracking individuals along this path, providing more clearly delineated patient populations. The criteria inform clinical diagnostic practice while supporting the identification of an “early,” non-clinical research population for studies to test the effectiveness of preventative therapies for AD (Lock 2013; Moreira 2017; Milne 2018). Further, the criteria (and a similar proposal from an International Working Group [Dubois *et al.* 2007]), introduce biological markers to diagnostic assessment for the first time. In doing so, they act as “trailblazers,” establishing a vector for research and clinical care which validates biomarkers’ potential for understanding an individual's relationship with (future) dementia, while also being “gatekeepers,” emphasizing that the potential of the technology itself has not yet been realized (Boenink 2018).

### The ill-charted territory of Alzheimer's genetics

The introduction of biomarkers to diagnosis reflects the growing role of biological indicators in the identification of Alzheimer's disease. For Lock (2005), the drive to biomarkers reflects a change in how biomedical research engages with the future, prompted by the uncertain predictive value and complexity of Alzheimer's disease genetics in comparison with single gene disorders. This is reflected in the focus within contemporary Alzheimer's disease research on the exploration of susceptibility genes and polygenic risk factors, even the most potent of which only contribute incrementally to an individual's lifetime risk of developing dementia. Further, the clinical introduction of genetic knowledge, notably within pharmacogenetics, has met significant resistance, not least because of socio-ethical concerns (Hedgecoe 2004).

The recognition of complexity is, of course, not new to post-genomic framings of Alzheimer's disease. Pathologist Martin Roth, in his closing summary to the first international symposium on Alzheimer's disease in 1969, pointed out that, while there was evidence of a hereditary component to Alzheimer's disease, “It is clearly unprofitable to think overall in terms of classical Mendelian phenomena, that is, in terms of major autosomal or sex-linked genes.” (Roth in Wolstenholme and O’Connor 1970, 301). Instead, he highlighted the “ill-charted territory” of “polygenetic” hypotheses. While his caution contrasted with the enthusiasm of others present,^1^ Roth's position reflected his focus on Alzheimer's disease as a condition seen and studied in the community, rather than the clinic, and its relationship with normal ageing, rather than as a homogeneous and clearly defined pathological entity (Moreira 2017). Between the caution of Roth and post-genomic complexity lies the experience of the molecular biology heyday in the 1980s and 1990s.

### The importance of the rare

Work on Alzheimer's disease in the late 1970s emphasized neurochemistry, focussing on deficits of the neurotransmitter acetylcholine and pursuing therapies that paralleled the apparent success of levodopa in the treatment of Parkinson's disease. Such work derived from the reconceptualization of dementia as a neurological disorder, which opened the possibility of a shared epistemic field and the transportability of knowledge (Moreira 2009). However, with the advent of molecular biological techniques, broadened diagnostic definitions, and new organizational structures for research, alternative epistemic referents came into focus.

Since the 1940s it had been known that people with Down's syndrome commonly developed symptoms of dementia in their 40s, that plaques and tangles were also found in the brains of those with this dementia, that this may provide insights into what was then “senile dementia” (Jervis 1948). These pathological relations of equivalence were translated into the terms of molecular biology as the amino acid sequence of the “beta-amyloid” protein found in plaques was elaborated (Glenner and Wong 1984). As a neurobiologist involved in neurotransmitter-focussed work on Alzheimer's disease in the 1970s later described, the sequencing of the amyloid protein was a pivotal moment:

From my perspective, the way AD research developed was that there was this whole “chemistry era,” which really predated me, and then ran through 1983, probably 1984, and then everything changed with the amyloid sequencing; and the whole field has gone mad since then. (Davies in Katzman and Bick 2000)

The sequencing provided a new form of evidence that the same protein was indeed found in both the brains of Alzheimer's disease patients and people with Down's syndrome – as authors Glenner and Wong described, they had provided:

[T]he first chemical evidence of a relationship between Down's syndrome and Alzheimer's disease. It suggests that Down's syndrome may be a predictable model for Alzheimer's disease. (1984, 1131)

The establishment of molecular relationships between Alzheimer's and Down's stabilized the value of molecular genetic studies of Alzheimer's disease, and created new intersections between the laboratory and the clinic. In the process, it generated new interest in conditions such as Down's syndrome, and rare familial forms of disease as “predictable models” of common disease. Writing in 1988, neuropathologist David Mann pointed out that the existence of equivalence suggested a population of “immense value” for research:

Younger patients with Down's syndrome may represent a model for study from which it may be possible to identify the site and nature of the earliest pathological changes of Alzheimer's disease itself. (Mann 1988)

Such models are situated along carefully delineated axes of similarity and difference, oriented towards a goal of prediction and control in a heterogeneous research and clinical field. The genetic and neuropathological promise associated with them reinforced the ongoing “quest” (Pollen 1996) to identify genes responsible for Alzheimer's disease. It resulted in the identification of a series of autosomal dominant mutations affecting the production or removal of amyloid, located in the genes presenilin 1 (PSEN1), presenilin 2 (PSEN2) and amyloid precursor protein (APP). Writing in the early 1990s, neurologist Daniel Pollen drew attention to the politics of scientific competition and collaboration associated with this race, the incorporation of familial knowledge into scientific data and the history and traditions and the movements of economic migrants and scientists in nineteenth and twentieth centuries Europe and North America that shaped Alzheimer's genetics research.

Research into the “Alzheimer's gene” established familial and early-onset Alzheimer's disease as a genetic condition – a distinctive genomic designation (Navon 2011) applied to the subset identified in the McKhann criteria, (and later retrospectively applied to Auguste D, the first case described by Alois Alzheimer [Keuck 2018]). However, as Pollen elaborated, the research was spurred, and wider interest generated, by the ability of this rare designation to speak to the common:

[I]n discovering the nature of the genetic defects in the less common early-onset form of familial Alzheimer's disease, scientists may perhaps also find the clues to the enigma of the far more frequent late-onset form of the disease which some have called “the disease of the century.” (Pollen 1996, 15)

Pollen here draws attention back to the connection between the familial and the frequent and the scalar promise of understanding what Clinton later described as the “genetic roots” of disease*.* They highlight the relationship between the “rare” and the “common” established through work on Down's syndrome and familial forms of disease. As a 1993 review put it “although the mutations are rare causes of the disease, they point to a general mechanism of aetiology and pathogenesis” (Lannfelt *et al.* 1993, 207).

## From genes to mice

The combination of genetic research, diagnostic reform and social movement established the existence of two scales of investigation, and the terms of connection between them. It provided the basis for the emergence of a bioclinical collective around AD which focussed attention and therapeutic hopes on the “amyloid cascade hypothesis” (ACH). The ACH was first outlined by John Hardy and David Allsopp, drawing on work led by Alison Goate from Hardy's group which had identified the first genetic mutations causing familial Alzheimer's disease – a missense mutation in the amyloid precursor protein on chromosome 21 identified in a UK family (V717I; the “London” mutation). They proposed that the hypothesis would form the basis for “rational design of drugs to intervene in this process” (Hardy and Allsop 1991, 383), and made efforts to start the commercialization of the discovery (Bubela, Vishnubhakat, and Cook-Deegan 2015). The ACH was subsequently re-described and elaborated in a review paper in *Science* by John Hardy and Gerald Higgins (1992). It proposed that the accumulation and deposition of beta-amyloid protein initiated a chain of molecular and cellular events resulting in the death of nerve cells and dementia. While the ACH continues to be contested and controversial (Lock 2013), the paper set it out in what Hardy later described as a “simple, clear and short” form which “even a venture capitalist or a corporate CEO can read to the end” (Hardy 2006). Such a clear, biologically defined trajectory establishing the ability of diagnose, predict and intervene, presented in an accessible fashion, represent, in many ways, the ideal – and idealized – output of molecular genetic research.

Hardy and Higgins’ paper represented the combination of two research strands in molecular biology. Higgins provided supporting evidence for Hardy's work on the APP gene in the form of a transgenic mouse model. Higgins and colleagues had overexpressed human DNA coding for a fragment of the amyloid precursor protein in the brains of mice. Their research concluded that this single change could lead to the neuropathological features observed in Alzheimer's disease. As Hardy described in 2006:

While I was visiting Gerry's lab, he and I had got on very well, and he showed me a manuscript he had in press in *Nature*, describing the production of transgenic amyloid mice with fulminant Alzheimer pathology, both plaques and tangles. He offered to show me slides from the mice, but he couldn't lay his hands on them at that time. I didn't mind because looking down a microscope is always a waste of time for me. However, the manuscript was stunning, and ostensibly described the full modeling of the disease process. (Hardy 2006, 152)

The mouse model described – published in *Nature* in 1991 – demonstrated that mice could mimic the biological changes seen in the brains of people with Alzheimer's disease, and which, unlike the “models” of trisomy 21 and familial disease, offered the possibility of experimentation. However, it subsequently became clear that Higgins’ model could not be replicated and that there were significant concerns about the validity of the findings. The *Nature* paper was retracted (Marx 1992), and with it, the initial animal evidence in favor of the hypothesis.

The development of transgenic animals that would offer a “good enough” model of Alzheimer's disease in terms of the neuropathological cascade, remained challenging. Indeed, Lannfelt's 1993 review, which included Hardy as senior author, raised the possibility that (in contrast to the human model diseases) animals may simply be unable to model Alzheimer's pathology and that while “[s]uch an assertion is as yet unanswerable” it “will indeed, be the explanation of choice if transgenic modeling experiments continue to fail.” (Lannfelt *et al.* 1993, 210).

### PDAPP mice

Attaining an appropriate alignment of mouse body and brain was critical to affirming the amyloid hypothesis, and make it useful to research. The first transgenic animal model of AD was first reported in 1995 by the Californian biotech company Athena Neurosciences, in collaboration with Eli Lilly (Games *et al.* 1995). The APPV717F mouse, also known as PDAPP, was the first transgenic model to provide a “robust” (Duff 2002) model of Alzheimer's disease pathology. PDAPP mice are genetically modified to reproduce a mutation found in the amyloid precursor protein (APP) gene in dominantly inherited forms of Alzheimer's disease. Specifically, the mouse models capture the V717F point mutation, a mutation which occurs at the same molecular site as the London mutation and was identified at the Indiana University School of Medicine (and thus termed the “Indiana” mutation; Murrell *et al.* 1991).

The development of the PDAPP mice prompted renewed excitement in the field – a commentary (again co-authored by Hardy) described how the transgenic animals represented both a source of therapeutic promise and stabilized understandings of the disease. It celebrated the availability of “these remarkable animals” (Duff and Hardy 1995, 476), and presented the mice as a conclusive moment in the amyloid story:

Workers investigating AD have been sharply divided into two camps: those who think amyloid deposition is central to the pathological process and those who do not … The work of Games and colleagues in making a mouse model of the disease settles this argument, perhaps for good. (Duff and Hardy 1995, 476)

As well as seemingly settling the biological debate, PDAPP mice reinforced the economic promise of AD research, as captured in a New York Times’ report describing the mice as “a rare piece of good news from the beleaguered biotechnology industry” (Fisher 1995, 8). Duff and Hardy advocated that “expanding and distributing colonies of the mice should be a priority” (Duff and Hardy 1995, 476). However, the challenges of breeding and circulating mouse models, and Athena and Lilly's efforts to capture the value from their research meant that the mice were initially used within those companies for therapeutic screening, before becoming widely available (Sedlak 1995).

Patents were submitted by Games and Athena to cover the transgenic models and their use for screening potential therapies, based on their greater similarity to naturally occurring Alzheimer's disease. Yet, like other models, PDAPP mice are “simultaneously like and unlike their human counterparts” (Lewis *et al.* 2013, 778); while they fail to reproduce aspects of AD pathology, notably the loss of neurons and neurofibrillary tangles, their ability to show amyloid plaques and sufficient behavioral and biochemical features of the disease positioned them as “good enough” representations of Alzheimer's disease. However, such models reify both behavioral tests and the centrality of genetics to the development of future treatments (cf Davies 2010).

### From mice to drugs

The PDAPP model is intimately connected with the humanized monoclonal antibody (MAb) therapy solanezumab, which entered development as a result of research with the mice conducted at Washington University in the 1990s. Monoclonal antibodies – standardized biological molecules produced in cell cultures – are an increasingly important part of both diagnostic and biopharmaceutical markets (Marks 2015). The research leading to solanezumab focussed on the development of a diagnostic test, specifically the use of murine antibody 266.2 (m266.2) to assess levels of beta-amyloid. By this point, the PDAPP strain was established as a widely used research tool, yet even here presented a site of unexpected resistance – as even within the homozygous PDAPP mice, only half of the mice displayed high levels of amyloid deposition (DeMattos *et al.* 2001).

The transformation of m266.2 from diagnostic to therapeutic molecule was described in the patent application lodged by Washington University and Eli Lilly:

We have now unexpectedly found that administration of the 266 antibody very quickly and almost completely restores cognition (object memory) in 24-month old homozygous transgenic mice (APPV717F). (Holtzman, DeMattos, and Bales 2002, no page)

While the description of surprise also provides narrative support for the claim of an inventive step worthy of patent protection, the patent describes the unexpected finding that antibody m266 did not simply attach to beta-amyloid, but drew it out from the brain, preventing toxic plaques forming and crucially, treating memory loss. It suggested the possibility of a novel “peripheral sink” (DeMattos *et al.* 2001, 8854) approach to the treatment of what the patent described as “conditions related to the Aβ peptide.” As this suggests, m266 is inextricably entangled with the claims and promise of the ACH – and indeed, the paper reporting the antibody's efficacy in mice started by recapitulating the hypothesis. However, as a contemporaneous review by Harvard neurologist Dennis Selkoe (2001) concluded, such drugs were also the missing piece in demonstrating the validity of the aetiological picture.

Six years after Hardy and Duff's celebration of the success of PDAPP mouse models in demonstrating the ACH, Selkoe positioned the launch of the first amyloid-focussed drug trials as simultaneously a test of the drugs and the hypothesis itself. However, as the story of solanezumab over the subsequent period shows, conducting this test has resulted in a continual realignment of drug and disease, and notably the relationship between the rare genetic forms of disease biologically recapitulated through animal models and the sporadic forms on which the large-scale social and financial promise of drug development rests.

### From mouse to human

The establishment of “good enough” mouse model of AD enabled the expanded study of genetic forms of Alzheimer's disease, while the model itself became black-boxed as a research tool (cf Nelson 2018) supporting the potential of m266.2. However, although the first successes in monoclonal antibody drugs came through the direct use of murine antibodies, most such molecules failed to translate directly into humans (Marks 2015). By the 1990s, the emphasis shifted to the development of “chimeric” and “humanized” antibodies, genetically reengineered forms with reduced immunogenicity primarily produced in mammalian cell cultures. Having thus “murinized” (a version of) Alzheimer's disease, human trials involved the “humanized” antibody – now named LY2062430 or solanezumab.

The first human clinical trials of solanezumab began in 2004, with Phase I trials conducted with people with mild to moderate dementia, with neat symmetry, at Indiana University (Siemers *et al.* 2010). This single site study with 20 people established the safety and tolerability of the drug in a human population, and provided the basis for an extension to a Phase II study, this time with 52 people across 6 sites (Farlow *et al.* 2012). The Phase II study completed in 2008, and Lilly immediately launched two large phase III studies. These studies, EXPEDITION and EXPEDITION2 – recruited a total of over 2000 people with mild-to-moderate Alzheimer's disease as defined by the McKhann criteria. The studies were conducted at 211 sites across 16 countries – the majority in North America and Europe, but also Brazil, Argentina, Poland, Russia, Japan, Korea, Taiwan and Australia, reflecting the global extension and geographical scaling of the solanezumab program (Henley *et al.* 2015). The conclusions from these studies were released in 2013, with the subsequent publication concluding bluntly that solanezumab “failed to improve cognition or functional ability” (Doody *et al.* 2014). Nevertheless, analysis of a subgroup of people with mild dementia suggested some effect on cognitive performance. It was also found that around 25% of the population diagnosed with Alzheimer's disease in each trial were “amyloid negative” (Karran and Hardy 2014). According to the revised diagnostic criteria discussed above (and in contrast to the McKhann criteria used for recruitment) they no longer fell within the Alzheimer's disease population.

These findings formed the basis for the third Phase III trial, EXPEDITION3. To capture the effect tentatively suggested in the first trials, this new study focussed entirely on people with mild dementia, and who were “amyloid positive.” As introduced earlier, this shifted the diagnostic ground while making this sub-group speak for the condition as a whole. In other words, it fragmented the common, in a move familiar within the worlds of “stratified” and “precision” medicine (cf Tutton 2014). Nevertheless, as described in the introduction, solanezumab again failed to show a significant effect in this population (Honig *et al.* 2018).

For sceptics, the results of the EXPEDITION studies, and particularly EXPEDITION3, cast serious doubts on the validity and value of the amyloid cascade hypothesis (Le Couteur, Hunter, and Brayne 2016). Nevertheless, as Lock puts it, “the amyloid hypothesis hangs on, battered and bruised though it is and remains the key postulate even as prevention moves to the fore” (Lock 2013, 216). Indeed, for proponents, the apparent ability of solanezumab to affect amyloid levels and the suggestion of possible small changes in cognition told quite the opposite story. In a 2016 commentary on the 25th anniversary of the ACH published before the EXPEDITION3 results, Hardy and Dennis Selkoe drew on the first two EXPEDITION studies, an unsuccessful trial of another MAb, crenezumab, and Phase I trial data from a further drug aducanumab. Reading from the secondary analysis of EXPEDITION and EXPEDITION2 which drove the launch of EXPEDITION3, they described “a probable signal in mild AD,” and reiterated the need to move “earlier” in the disease (Selkoe and Hardy 2016). Similarly, in his comments to the CTAD symposium on EXPEDITION3, Paul Aisen, the head of the NIA Alzheimer Disease Cooperative Study argued that “we have a negative study that confirms a beneficial treatment” and which was “not the refutation of the amyloid hypothesis, it's the confirmation of the amyloid hypothesis – the strongest evidence we have to date” (author notes). For Aisen also, EXPEDITION3 supported a continuing move to intervene earlier.

### From people to genes to people

The failure of solanezumab, and of the field in general to develop a disease modifying drug has been narrated as a problem of doing too little, too late in the disease process, and in terms of the limitations of animal models such as PDAPP to provide a solid basis for therapeutic scaling. As pharmaceutical company researcher Hans Mobius set out in a reflection on the prevalence of negative results in Alzheimer's disease clinical trials, animal models are central to this problem:

To date, there have been more than 200 clinical development failures for various reasons and all completed Phase III trials on disease modifying compounds in AD during the past decade have failed to demonstrate a cognitive or clinically relevant improvement. Of even greater concern are the discrepancies between the positive results seen in animal models, e.g. removal of brain amyloid and failure to show benefit in clinical trials when targeting the same mechanism. Despite major investments and increased understanding of pathophysiology, proof of disease modification remains elusive today. (Möbius 2013)

For Mobius at least, existing animal models were no longer “good enough” for their purpose, as the geographies of corporeal equivalence between animal and human manifestations of Alzheimer's disease start to fragment:

There are no animal models of AD that meet **all** human disease criteria—so **there is no actual model of AD**. (Möbius 2013; emphasis added)

Similarly, at the agenda-setting 2012 National Institutes of Health/National Institute on Aging “Alzheimer's Disease Research Summit,” one contributor suggested that “we need to ask, what have we actually been testing in our preclinical animal studies?” (Greenberg in NIH 2012, no page). The move to focus on earlier disease stages and disregard animal models delimits the space of failure within the research system, to the space of the relations between species and scales, and temporally, to the stage of disease. Potential success then lies in reconfiguring relationships of equivalence and engaging with new temporalities of Alzheimer's disease, notably through the ability to predict and pre-empt.

The focus on prediction and pre-emption repositions genetics at the heart of biotechnological promise – not as genomic technology, but as a “pre-genomic” biology which is predictable, potentially more amenable to control, and human. This is captured in the “third wave” of Phase III trials of solanezumab. Where the first (EXPEDITION1 and EXPEDITION2) and second wave (EXPEDITION3) of trials edged back towards the earliest symptoms of dementia, ongoing trials move into the realm of prediction and “secondary prevention” (Carrillo *et al.* 2013).

Here I turn to fieldnotes from a second Alzheimer's conference, held in London in the summer of 2017. The notes derive from a satellite meeting to the main conference, sponsored by a leading pharmaceutical company and held in the evening in the basement of a city-centre hotel. Such meetings provide conference attendees a means of hearing in a smaller setting from some of major names in the field, and provide a venue for such leaders in the field – and companies – to stake claims to the future direction of Alzheimer's disease research.

The first speakers set out the rationale, and current knowledge related to the prevention of Alzheimer's disease dementia. Bruno Dubois, professor of neurology in Paris, describes his work proposing revisions to diagnostic criteria for Alzheimer's disease. The second speaker, Colin Masters, contributed to much of the early work on both the molecular genetics of Alzheimer's disease and the ACH, and now leads a major brain imaging initiative in Australia. Together, they orient and frame the current state of research and outline the relationship between biomarkers, trials and the future clinic.

The other speakers present their respective leadership of the two remaining Phase III clinical trials of solanezumab. Reisa Sperling, who led the NIA-AA group to develop criteria for research in “preclinical” Alzheimer's disease, describes the Anti-Amyloid in Asymptomatic Alzheimer's disease trial (A4), a partnership between the US National Institute of Ageing and the pharmaceutical company Eli Lilly. The A4 study extends the approach adopted in EXPEDITION3. However, rather than supplementing cognitive symptoms with biological measures as criteria for trial inclusion, it replaces them entirely – the trial recruits only people without cognitive complaints but who are “amyloid positive.” The fourth speaker, Randall Bateman, leads the “trials unit” of the Dominantly Inherited Alzheimer's disease Network, or DIAN. DIAN is an international network of families affected by familial forms of disease. Like A4, DIAN is a public-private partnership of the type which dominates the Alzheimer's research ecosystem, supported by governments through programs like the US National Alzheimer's Plan Act (NAPA) or the EU Innovative Medicines Initiative (Milne 2018).

Bateman describes a second trial occurring alongside the A4 study of “asymptomatic” individuals, the DIAN trials unit (DIAN-TU) study with people with “presymptomatic” Alzheimer's disease. In the ground of this delicate lexical distinction between the two studies lies a key boundary between sporadic, late onset Alzheimer's disease and the familial, early-onset form, and the source of scientific and economic promise. DIAN-TU differs significantly from the A4 and EXPEDITION studies, most notably in order of magnitude. While the latter recruit research subjects in the thousands, DIAN-TU's total population is around 300. However, in the move from asymptomatic to presymptomatic, amyloid biomarkers are re-connected with genes, and the latter's association with prediction and control re-emphasized. Bateman illustrates this by displaying the dominant image of contemporary AD research, the so-called “Jack curves,” a hypothetical model of biological and cognitive changes associated with Alzheimer's disease and named for lead author Clifford Jack. The curves posit a temporal sequence of biological and cognitive processes prior to the development of dementia – crystallising what the DIAN group elsewhere refer to as the “pathochronology” of Alzheimer's disease (Morris *et al.* 2012, 975).

Bateman showed that the longitudinal study within DIAN had provided empirical data to illustrate the hypothetical sequence. He set out the importance of this data in a co-authored editorial published shortly before the conference, echoing Mann's discussion of the predictability of genetic conditions to argue that such research “provides researchers with a suite of biomarkers to test the efficacy of treatments after three to five years.” Critically, the authors continue, “that's a time frame that is likely to be palatable to those funding trials such as pharmaceutical companies” (McDade and Bateman 2017, 154). As discussed elsewhere, Alzheimer's disease research is continually forced to grapple with the problematic temporalities of ageing (Lock 2013; Milne 2016). For researchers and companies, studies such as DIAN-TU offer the potential to save both time and money. Bateman's description mobilizes a genetic imaginary which emphasizes the ability of genetic knowledge to make Alzheimer's disease manageable, and as he continues, to be *scaled*:

[E]ven for people with different mutations (230 are known), amyloid plaques are a common target to focus on — one that is shared with the much more common late-onset Alzheimer's disease. (McDade and Bateman 2017, 155)

The paper positions amyloid as the pivot between rare and common forms of disease, and establishes the promise of research in the former in relation to the latter. It reflects the persistence of an imaginary of genetic promise that has continuities with 1980s molecular biology.

Further, while Bateman's framing presents the value of genetic forms of AD in terms of shared pathology, a second position emphasizes the “translational” role of this population, as described by two clinical triallists:

The conundrum is that we use transgenic animal models based on the amyloid hypothesis to test compounds for efficacy, and subsequently we exclude the patients whose pathomechanism is closest to the model organism, in which it is most likely that the observed effect is replicated. (Szigeti and Doody 2011, 3)

This article, co-authored by Rachelle Doody, a lead investigator on the EXPEDITION solanezumab trials, is a call for greater inclusion of people with early onset and genetic forms of Alzheimer's disease in clinical trials. It reflects the fact that, over much of the last 30 years, familial forms of Alzheimer's disease have received comparatively little therapeutic study. Clinical trials frequently exclude people with dominantly inherited forms of disease – primarily through inclusion criteria that restrict study entry by age. In their discussion, Skigeti and Doody draw attention to the inconsistency in this approach, given the basis of animal models in genetic forms of disease, and, we can add, the basis of drug development in these animal models.

Autosomal dominant forms of Alzheimer's disease are thus positioned by researchers working on both familial and sporadic disease, and by regulators, as an increasingly important locus of drug development. This represents however, a recalibration of the relationship between familial and sporadic disease, or the rare and the common. Over the past 25 years, biological understandings of Alzheimer's disease gleaned from genetic mutations have proved repeatedly resistant to manoeuvres to scale them into the wider population with dementia. The focus on amyloid provides a mechanism for aligning and bridging disease scales. However, it has consequences for the rare disease – drawn more closely into the orbit of the common – and the common disease, defined in terms that most closely associated it with the rare.

## Geneticization, calibration and translation

I have argued that the role of genetics in Alzheimer's disease drug development suggests the continuing importance of a genetic imaginary in shaping the future of research and therapy for the condition. This imaginary underpins the amyloid model that continues to dominate drug development, and shapes the contours of the relationship between forms of disease that are considered as rare and common. In doing so, it provides the basis for the promise of scale. I have described an epistemological and ontological transition from the focus on molecular genetics as the etymon of disease, through the development of mouse models to the common and complex. Through these processes, the imagined potential of genetics for drug development in AD emerges from its ability to establish the molecule and the population as scales which are biologically, but also economically, complementary, and between which concepts and objects such as drugs can move.

The relationship described between the scales associated with rare and common forms of Alzheimer's disease as they are constructed around solanezumab has relevance for other domains, including those presented as analogical referents. It suggests that discussion of the genetic imaginary (Weiner *et al.* 2017), can be extended by considering how this imaginary represents a structuring framework for a process of translation and scaling through which the rare speaks for the common, and which provides the route to realizing the economic and social potential of genomics.

Scaling genetic understandings through the materials and bodies of biomedical translation, however, privileges those elements of a system that are most amenable or least resistant (cf Tsing 2012). Such elements consequently move to become central nosological as well as aetiological features of disease, as Alzheimer's disease comes to be considered in terms of its proteins rather than its symptoms. Moreover, the relations of scale and equivalence established around pivot points such as amyloid require constant maintenance and re-assembly. This work of extension, maintenance and calibration (Lewis *et al.* 2013) establishes the parameters of what constitutes “good enough” relationships, not only between human disease and animal models, but between forms of human disease. As introduced above, work on cholinesterase inhibitors was based on the apparent transportability of knowledge from equivalent research on Parkinson's disease. It involved an essentially analogical relationship which posited that the two diseases corresponded in important molecular aspects. In a sense, this represents a classic ANT translation, that is the creation of “convergences and homologies by relating things that were previously different” (Callon 1980, 211). As Knorr pointed out in an early analysis of analogical reasoning in science, such “creative extension[s] of knowledge” are important in establishing “the promise of success” (1980, 37). However, as she continued, the instability of such relationships means that “much of … scientists’ work goes into demonstrating why and to what degree some object is or is not an instance of a certain kind” (Knorr 1980, 29), identifying and extending interpretations, demonstrating their relevance and producing and reproducing the conceptual and material similarities through which extensions are stabilized. Thus, despite a long practical history of working with rare and common forms of Alzheimer's disease together, the relationship between genetic and sporadic disease captured in the ACH has required repeated re-iteration and maintenance – a process that John Hardy described in frustration as like Procol Harum being repeatedly asked to sing “A Whiter Shade of Pale” (Hardy 2006, 152). However, Knorr's conclusions about the limits of such relationships suggest the problems of the translation and scaling associated with the ACH. Critically, Knorr suggests, the projects and definitions of success associated with such maintained relationships involve:

[A] path of known destination on which they have a good chance to arrive at their goals, and on which they have a good chance to arrive there first or fastest. (Knorr 1980, 40)

Consequently, she continues “[a]nalogical transfers form a firm ground for controlled rather than uncontrolled risks” (Knorr 1980, 40). Similarly, Callon describes how the building of convergences and homologies both defines and delimits “the identity of actors, the possibility of interaction and the margins of manoeuvre” (Callon 1984, 203). Such solutions do not open the possibility of surprise – in the terms introduced by Shostak *et al.* above, they create a form of path dependency which provides “conservative” (Knorr 1980) strategies and results in the solidification or “petrification” of what counts as true. By basing drug development on a model constructed around relationships between the rare and the common, research follows a path of least resistance in order to establish a promise at scale. In contrast, following Tsing (2012), in “salvaging” therapeutic research into Alzheimer’ disease there may be value in pursuing the sites at which resistance to scaling occurs, and at which convergence and homology collapse.

## Note

1 Not least geneticist Richard Pratt, who commented at the meeting that “[t]he closer one gets to the gene -and that means on the biochemical level – the more likely one is to get the right answer” (Pratt in Wolstenholme and O’Connor 1970, 141).
